# Development and Characterization of Synthetic Glucopyranosyl Lipid Adjuvant System as a Vaccine Adjuvant

**DOI:** 10.1371/journal.pone.0016333

**Published:** 2011-01-26

**Authors:** Rhea N. Coler, Sylvie Bertholet, Magdalini Moutaftsi, Jeff A. Guderian, Hillarie Plessner Windish, Susan L. Baldwin, Elsa M. Laughlin, Malcolm S. Duthie, Christopher B. Fox, Darrick Carter, Martin Friede, Thomas S. Vedvick, Steven G. Reed

**Affiliations:** 1 Preclinical Biology, Infectious Disease Research Institute, Seattle, Washington, United States of America; 2 Initiative for Vaccine Research, World Health Organization, Geneva, Switzerland; 3 Immune Design Corp., Seattle, Washington, United States of America; Southern Illinois University School of Medicine, United States of America

## Abstract

Innate immune responses to vaccine adjuvants based on lipopolysaccharide (LPS), a component of Gram-negative bacterial cell walls, are driven by Toll-like receptor (TLR) 4 and adaptor proteins including MyD88 and TRIF, leading to the production of inflammatory cytokines, type I interferons, and chemokines. We report here on the characterization of a synthetic hexaacylated lipid A derivative, denoted as glucopyranosyl lipid adjuvant (GLA). We assessed the effects of GLA on murine and human dendritic cells (DC) by combining microarray, mRNA and protein multiplex assays and flow cytometry analyses. We demonstrate that GLA has multifunctional immunomodulatory activity similar to naturally-derived monophosphory lipid A (MPL) on murine DC, including the production of inflammatory cytokines, chemokines, DC maturation and antigen-presenting functions. In contrast, hexaacylated GLA was overall more potent on a molar basis than heterogeneous MPL when tested on human DC and peripheral blood mononuclear cells (PBMC). When administered *in vivo*, GLA enhanced the immunogenicity of co-administered recombinant antigens, producing strong cell-mediated immunity and a qualitative T_H_1 response. We conclude that the GLA adjuvant stimulates and directs innate and adaptive immune responses by inducing DC maturation and the concomitant release of pro-inflammatory cytokines and chemokines associated with immune cell trafficking, activities which have important implications for the development of future vaccine adjuvants.

## Introduction

The goal of immunization is to generate specific and strong immune responses against target antigens with minimal side effects. In the past, most vaccines have been developed using live attenuated or killed whole organisms that do not require adjuvants, in some cases because they mimic natural infection and inherently contain microbial immune modulators, including TLR ligands. Subunit vaccines, consisting of a pathogen protein with an immunostimulatory adjuvant, offer a distinct advantage over live attenuated or heat killed vaccines, in that they do not have the potential to be infectious, and therefore can be administered to immunocompromised, as well as immunocompetent people. However, unlike attenuated live vaccines, many subunit vaccine antigens require adjuvants to enhance the strength and duration of the immune response to these antigens which may be weakly immunogenic on their own [1], [2], [3]. Until recently, salts such as aluminum hydroxide and aluminum phosphate (alum) were the only adjuvants in approved human vaccines in the USA. Although alum is effective in boosting antibody responses, achieving these responses requires repeated administration and tend to be biased towards T helper (T_H_) 2, rather than T_H_1, immunity [4]. However, effective vaccines against some of the major causes of death worldwide (HIV, tuberculosis, malaria and possibly influenza) require both humoral and T cell responses, particularly T_H_1-mediated immunity. As a consequence, there is much effort devoted to developing adjuvants that can promote protective immunity through induction of enhanced and durable antibody and T_H_1 responses.

It has long been known that LPS, contained in the outer membrane of Gram-negative bacteria, is a potent stimulator of the immune system, albeit toxic. A non-toxic derivative of LPS, monophosphoryl lipid A (MPL®), has been produced from *Salmonella minnesota* R595 by removal of the core carbohydrate group, the phosphate from the reducing-end glucosamine, and the acyl chain from the 3′-position of the disaccharide backbone [5]. The resulting product demonstrates only ∼0.1% of the inflammatory toxicity of its parent molecule, LPS [6], [7]. Furthermore, it promotes the differentiation of IFN-γ secreting T_H_1 CD4+T cells, and enhances immune responses to co-administered antigens. MPL® combined with alum (AS04) is the adjuvant contained in two approved vaccines (Cervarix®, and Fendrix®, both produced by GlaxoSmithKline Biologicals (GSK), for human papilloma virus and hepatitis B virus, respectively). GSK has also developed liposomal (AS01) and oil-in-water (AS02) formulations containing MPL® that are included in malaria [8] and tuberculosis [9] vaccines that are currently in clinical trials. In addition, we are evaluating MPL® in a stable oil-in-water emulsion (MPL®-SE) as part of a leishmaniasis vaccine [10], [11].

Detection of microbes by the mammalian immune system is mediated by several families of pattern-recognition receptors, including TLRs, and this recognition leads to the activation of innate and adaptive immune responses [12]. TLRs, of which ten are known to be functional in humans, are linked to the control of bacterial and viral infections through one or more adaptor proteins containing a Toll–interleukin 1 receptor domain. TLR4, targeted by LPS and related molecules such as MPL, is unique among the TLR as it induces two distinct signaling pathways controlled by (i) MyD88/MAL which results in activation of nuclear factor (NF)-κB for induction of a number of NF-κB-dependent genes and inflammatory cytokines, and (ii) TRIF/TRAM which induces production of type I interferons (IFN) (reviewed in [13]). TLR4 also plays a non-redundant role in eliciting DC maturation which is key in adjuvant-mediated vaccine-antigen immunogenicity, since DC play a significant role in priming naïve T cells and initiating potent immune responses [14]. Recent studies have demonstrated that both MyD88 and TRIF synergize for maximal DC activation [15].

In this study, we explored the advantages of a pure synthetic hexaacylated lipid A derivative, glucopyranosyl lipid A (GLA), over naturally-derived and more heterogenous MPL [16], [17], [18], [19]. We characterized MyD88- and/or TRIF-dependent gene transcription profiles and kinetics induced by GLA on mouse and human DC, and correlated mRNA transcript with protein levels. In addition, we present data on the ability of GLA to induce systemic innate responses in vivo, and promote T_H_1 responses to co-administered antigens.

## Materials and Methods

### Reagents

GLA (molecular weight 1763.5) was synthesized by Avanti Polar Lipids (Alabaster, AL) or Albany Molecular Research, Inc. (AMRI, Albany, NY). Purified detoxified lipid A (MPL, *Salmonella minnesota* R595) (molecular weight 1525.1) was purchased from Avanti Polar Lipids. LPS (*Salmonella minnesota* R595) was purchased from Sigma-Aldrich (St Louis, MO). The structure and molecular characteristics of GLA have been described previously [20].

### Mice

Female C57BL/6 and BALB/c mice were purchased from Charles River (Wilmington, MA) or Jackson Laboratories (Bar Harbor, ME). Mice (6–12 wk of age) were maintained under pathogen-free conditions in the animal facility at IDRI, and treated in accordance with the guidelines of the IDRI Animal Care and Use Committee.

### Adjuvant Formulations

GLA and MPL were formulated as aqueous suspensions (AF) as previously described [20]. Purity was assessed on samples at a concentration of 50 µg/mL by high-pressure liquid chromatography (HPLC) and mass spectrometry [21]. Mass spectrometry data was collected by electrospray ionization in negative ion mode on a Fourier Transform Ion Cyclotron Resonance Mass Spectrometer (Bruker APEX Qe 47e). GLA-SE was prepared by mixing GLA with the squalene oil phase (SE) [21] before mixing with the aqueous phase and processing as previously described [20]. The stock formulation contained GLA at 1 mg/mL in 10% oil and was filter sterilized with a Millipore Steripak GP10 filter unit. GLA and MPL formulations were tested for stability by Dynamic Light Scattering (DLS) using a Malvern Zetasizer Nano [21]. Stability testing was done for the following time points: Day of Manufacture (DM); 1 and 2 wks; 1, 3, 6, 9 and 12 months. Concentration testing was also done on GLA-SE by HPLC [20] and found to be consistent through the formulation procedure (data not shown).

### Cells

#### Mouse DC

Bone marrow-derived DC (BMDC) were generated *in vitro* from the aspirated bone marrow from the femurs of mice. Single cell suspensions were prepared, washed and suspended at a concentration of 1×10^6^ cell/mL in complete medium (RPMI 1640, 10% fetal bovine serum, 1% L-glutamine, 1% penicillin/streptomycin) supplemented with 20 ng/mL recombinant mouse GM-CSF and 20 ng/mL IL-4 (PeproTech, Rocky Hill, NJ). On day 3, DC were supplemented with 10 mL of complete medium and GM-CSF. The non-adherent cell population was used in experiments at day 5–7.

#### Human DC

Informed consent was obtained from all the subjects and the study was approved by Western IRB, Seattle, WA. Buffy coats were obtained from healthy volunteers and PBMC were isolated by standard Ficoll-Hypaque density gradient centrifugation. DC were generated from PBMC following either adherence on plastic or CD14+ positive selection following standard methods which are briefly outlined below. Monocyte-derived DC were generated by plastic-adherence of PBMC (15×10^6^ cells/well) for 1 h in RPMI medium containing 1% fetal bovine serum. After incubation, non-adherent cells were removed by three washes with 1x PBS and the remaining adherent cells were then cultured in complete medium supplemented with GM-CSF (50 ng/mL) and IL-4 (20 ng/mL) (both Invitrogen, Carlsbad, CA). On day 3, the DC cultures received a supplemental dose of GM-CSF and IL-4. Non-adherent and loosely adherent DC were collected and used in the experiments after 5–7 days in culture. Alternatively, CD14+ monocytes were magnetically labeled with CD14 MicroBeads (Miltenyi Biotec, Auburn, CA) and purified on a MACS separator following the manufacturer's protocol. The eluted fraction containing the CD14+ cells were cultured at 0.5–1×10^6^ cells in complete medium containing GM-CSF (50 ng/mL) and IL-4 (50 ng/mL) for 5 days prior to use in the experiments. DC preparations contained ∼95% DC, no CD14+ cells and <2% contaminating B cells as assessed by morphology and FACS analysis (data not shown).

### Gene Expression in Response to TLR4 Agonists

#### Microarray

Cultured mouse BMDC (5×10^5^) were treated with buffer, or TLR4 agonists GLA (0.5 µg/ml), MPL (0.5 µg/mL), and LPS (40 ng/mL) for 4 h at 37°C. Total RNA was extracted with an RNeasy kit (Qiagen, Valencia, CA), amplified and biotinylated using the MessageAmp II aRNA amplification kit (Applied Biosystems, Warrington, UK), hybridized to the mouse inflammation 4x2K CustomArray (Combimatrix, Mukilteo, WA), and scanned with a GenePix Scanner (Molecular Devices, Sunnyvale, CA). Comparative analysis of treated versus untreated samples was done using BRB-ArrayTools software (NCI). Statistical significance was determined by a two-sample *t*-Test and a p-value <0.001.

#### Branched DNA assay

Mouse BMDC and human monocyte-derived DC (5×10^4^) were treated with 1 µg/mL of the TLR4 agonists MPL, GLA and LPS, or buffer alone (control) for 2–8 h at 37°C as specified in the legends. Cells were lysed and mRNAs for GAPDH, IL-1α, IL-1β, IL-6, IL-12p40, TNF, CCL2, CCL3, CCL4, CCL5, CCL7, CXCL1, CXCL10, IFN-β, IFIT-1, MX1, PKR, G-CSF, ICAM-1, CD40, CD80, CD83, and/or CCR7 were captured using the QuantiGene multiplex assay (Panomics, Fremont, CA) according to the manufacturer's instructions. Transcript levels were quantitated using the Luminex 200 system (LuminexCorp, Austin, TX) and analyzed using the MasterPlex EX software (Miraibio, San Francisco, CA).

### Cytokine/Chemokine ELISA

Mouse and human DC, and human PBMC were seeded at 2×10^5^ cells/well in complete RPMI medium, and stimulated for 4–24 h at 37°C with 0.001–1000 ng/mL of the TLR4 agonists as indicated in the figure legends. Supernatants were collected and analyzed for IL-1β, IL-6, IL-12p40, TNF, CCL4, CCL5, CCL7, CXCL10, and/or IFN-β. These cytokine and chemokine levels were determined by sandwich ELISA using paired antibodies (eBioscience, San Diego, CA, BD Biosciences, San Jose, CA, or Invitrogen), or by using a custom Luminex-based multiplex immunoassay kits (Procarta Cytokine Assay Kit: Affymetrix, Santa Clara, CA). Briefly, culture supernatants were incubated with polystyrene beads coated with antibodies corresponding to the different cytokines, and developed according to the manufacturer's instructions. Bead size and fluorescence were measured on a Luminex 200 and data was analyzed using the Masterplex QT software (Miraibio).

### Cell Marker Expression and Flow Cytometry

DC were incubated with 1–1000 ng/mL of TLR4 agonists for either 24 h to determine DC cytokine expression or 48 h to analyze DC surface marker expression, unless otherwise stated. For TLR blocking experiments, DC were pre-incubated for 1 h at 37°C with 1 µg/mL of anti-TLR-2 (clone TL2.1), anti-TLR4 (clone HTA125), or IgG2a isotype control (clone eBM2) (eBioscience). Briefly, DC maturation was determined by using fluorochrome-conjugated antibodies that recognize following surface molecules: CD11c, MHC Class II, CD86, and CD40 (mouse, eBioscience) and CCR7, CD80, CD83, CD86, and CD40 (human, eBioscience). DC were then fixed with Fix buffer (BD Biosciences) and analyzed by flow cytometry or permeabilized and further incubated with fluorochrome-conjugated antibodies for intracellular cytokine (eBioscience). DC were characterized by FSC vs SSC followed by gating on CD11c and MHC class II expression. Data were collected on an LSRII flow cytometer and expression of various markers was assessed using FlowJo (TreeStar, Ashland, OR) or FACSDiva™ (BD Biosciences) analysis software.

### T cell Stimulation Assay

BMDC (5×10^4^) from C57BL/6 mice were incubated for 48 h with 50–5000 ng/mL ID83 plus either media, or aqueous GLA or MPL at 1, 5 and 20 ng/mL, and CD4+ T cells (2×10^5^) from splenocytes of ID83-immunized mice purified over MACS column (Miltenyi) following the manufacturer's protocol. Supernatants were analyzed for IFN-γ by ELISA (eBioscience).

### Systemic Effects of Adjuvant Formulations

C57BL/6 mice were injected intramuscularly (50 µL per quadriceps) three times, two weeks apart with 8 µg ID83 [22] antigen either formulated with GLA-SE (1, 5 or 20 µg), MPL-SE (1, 5 or 20 µg) or SE alone.

#### Serum proteins

Sera were collected at time 0 and 4 h following the first injection and tested for the presence of IL-12p40, TNF, IL-6, MCP-1, CCL5, CXCL10 by multiplex ELISA as described above.

#### T helper cell priming

Seven days after the last immunization, splenocytes were plated at 2×10^5^ in complete medium, and incubated for 72 h with 10 µg ID83. Supernatants were collected and analyzed for IFN-γ cytokine production by ELISA (eBioscience).

### Statistical Analysis

Statistical analysis of the data was performed using the Student *t* Test using GraphPad Prism version 4.0 for Windows (GraphPad Software, San Diego, CA).

## Results

### Formulations of Synthetic Glucopyranosyl Lipid Adjuvant

**Figure 1:**
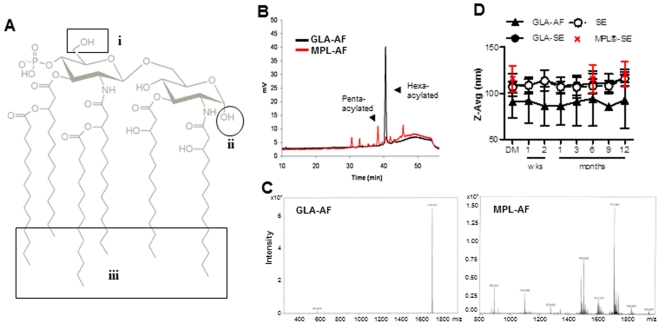
Stability of GLA adjuvant formulations. **(A) Differences between the synthetic GLA (structure in grey) and naturally occurring endotoxins. (i) – The GLA has no attached residues on the hydroxyl; endotoxins have some or many sugars attached to this site. (ii) – There is no second phosphate on GLA; naturally occurring lipid A cores have two phosphates with one attached to site “ii”. (iii) – The chain attachment positions, number, and lengths are defined. Naturally occurring species can have a variety of attachment sites, number of chains and the number of carbons within the chains vary. (B)** HPLC chromatograms of GLA and MPL. Arrows indicate the predominant acylated form: hexa- (GLA) and pentaacylated form (MPL). (**C**) Mass spectrometry profiles of GLA and MPL. (**D**) Particle size over time of GLA-AF and GLA-SE. As controls, stability of -SE and MPL-SE are shown. Data presented are the mean ± SD of 3–4 different lots of adjuvant formulations.

GLA is a synthetic lipid A derivative composed of a disaccharide backbone, a single phosphate group, and six C_14_ acyl chains [**20**] **(** **Fig. 1A** **). Several differences exist between synthetic GLA and naturally occurring endotoxins including (i) the absence of attached residues on the hydroxyl, (ii) the absence of a second phosphate on GLA, and (iii) defined position, number and lengths of chain attachments and carbon number (** **Fig. 1A** **). Naturally occurring species can have a variety of attachment sites, number of chains and the number of carbons within the chains vary.** HPLC profile of GLA confirmed the presence of a single peak corresponding to the hexaacylated form of the molecule by HPLC (Fig. 1**B**) and mass spectrometry (Fig 1**C**). In contrast, MPL showed one major peak corresponding to the pentaacylated form (Fig. 1**B–C**), a minor peak corresponding to a hexaacyl form, and three additional minor peaks with higher and lower molecular weight illustrating the more heterogeneous composition of MPL. GLA and MPL were prepared as an aqueous (-AF) or oil-in-water stable emulsion (-SE) formulations; **GLA in the SE formulation elicits more robust T_H_1 immune responses** [22], [23], [24], [25], [26****] **. However, the oil-content prohibits the use of the SE formulation for** ***in vitro*** **evaluation, therefore GLA was formulated and tested** ***in vitro*** **in an aqueous formulation.** GLA-AF and GLA-SE showed particle sizes averaging 89.7±3.4 and 110.7±3.0, respectively (Fig. **1D**). In comparison, lots of MPL-SE averaged 117.2±2.7.

### BMDC Responses to Synthetic Glucopyranosyl Lipid Adjuvant GLA

**Figure 2:**
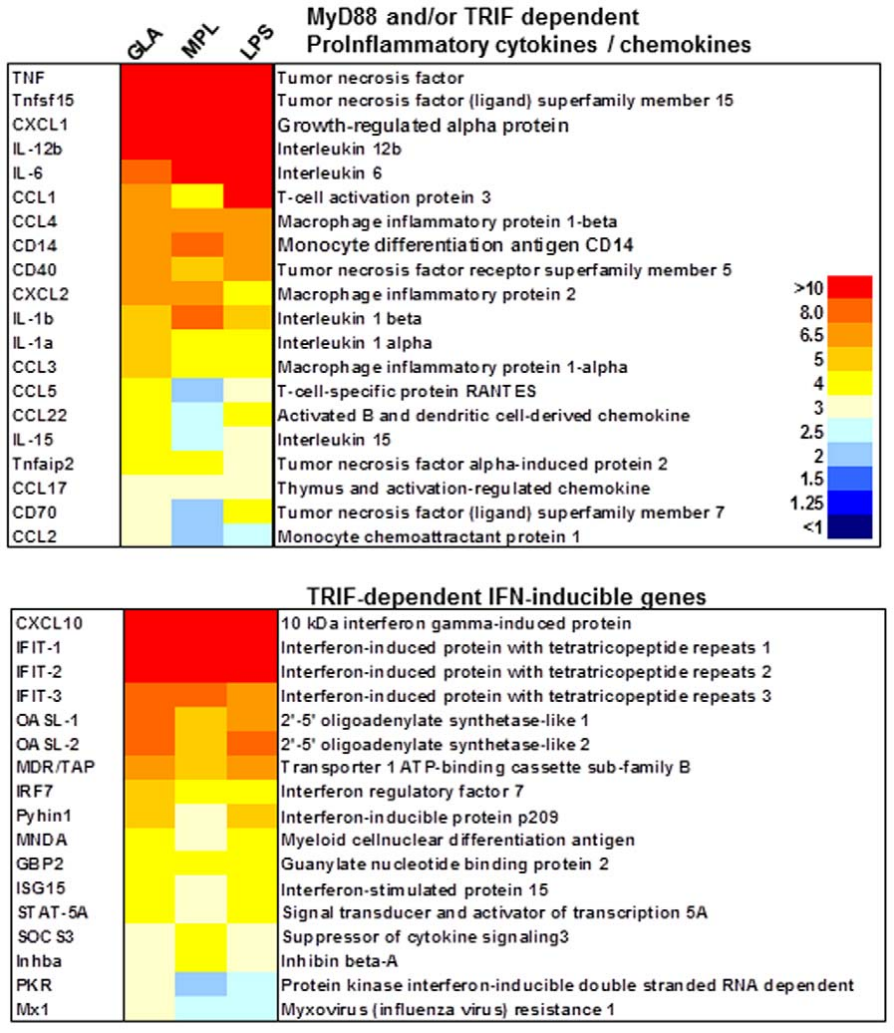
BMDC gene expression in response to TLR4 agonists. BMDC were stimulated for 4 h with GLA-AF (0.5 µg/mL), MPL-AF (0.5 µg/mL), or LPS (40 ng/mL). Total RNA was extracted, amplified, biotinylated, and hybridized to the mouse inflammation 4x2K CustomArray (Combimatrix). Comparative analysis of treated versus untreated samples was done using BRB-ArrayTools software (NCI). Statistical significance was determined by a random-variance *t*-test and a p-value <0.001. A 2.5-fold change above untreated samples was considered significant upregulation. Selected genes were partitioned into distinct categories and displayed in the heat-map for each set.

Adjuvants activate innate immunity by inducing cytokine and chemokine expression by DC and participate in the maturation of these cells [27], [28]. We recently reported that murine RAW264.7 and human MM6 monocyte/macrophage cell lines responded to synthetic lipid A stimulation *in vitro* by secreting TNF, IL-6, and CXCL10/IP-10 [20], [23], in a TLR4 dependent fashion [23]. To extend our understanding of the genes stimulated by GLA, and in comparison to other TLR4 ligands, BMDC were stimulated for 4 h and the isolated mRNA were amplified and hybridized to a custom mouse inflammation array (CombiMatrix) containing 840 genes. Global normalization was used to median the center of the log ratios on each array followed by random-variance t-test between treated samples (TLR agonist stimulated) and untreated samples (buffer control). Genes were considered significantly upregulated in treated samples if their p value was less than 0.001 and whose expression was at least 2.5-fold above untreated samples. RNA transcripts for pro-inflammatory cytokines, chemokines, and corresponding inducible genes, particularly TNF, IL-6, IL-12β, were up-regulated by GLA and MPL stimulation when compared to buffer control. RNA transcripts for the TRIF-dependent type I interferon-inducible genes and transcription factors were also upregulated (Fig. 2). Kinetic studies were further performed on a subset of 8 genes that are activated by the MyD88 and/or TRIF pathway (Fig. S1, upper panel) and 6 genes activated through the TRIF pathway only (lower panel). At the agonist concentration tested (1 µg/mL), GLA and MPL showed similar transcript levels and kinetics for all the RNA tested. Responses to stimulation with equivalent concentration of LPS were higher for all the MyD88- and/or TRIF-dependent activated genes tested, with the exception of CCL3 and CCL4 (similar levels). TRIF only-dependent genes showed similar transcript levels and kinetics (4 h and 8 h, but not at 2 h) in response to LPS compared to GLA stimulation, with the exception of G-CSF which showed increased levels of RNA transcripts.

**Figure 3:**
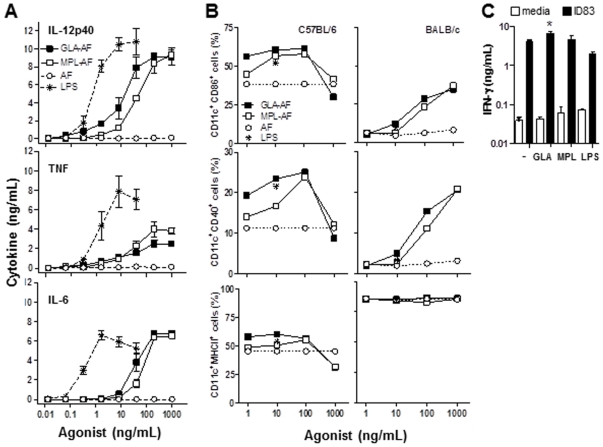
Dose-dependent activation and maturation of BMDC in response to GLA stimulation. BMDC were stimulated with 0.01–1000 ng/mL of GLA-AF, MPL-AF, LPS or equivalent volumes of AF. (A) IL-12p40, TNF, and IL-6 cytokine levels in culture supernatants after 24 h. (B) Percentage of CD86^+^, CD40^+^, and MHC Class II^+^ DC cells determined within the CD11c^+^ gate after 48 h of incubation with the TLR4 agonists (C57BL/6, left panels; BALB/c, right panels). (C) DC were pulsed with media or ID83 antigen (50 ng/mL), without or with 1 ng/mL of TLR4 agonist, washed and further incubated with antigen-specific CD4 T cells for 48 h. IFN-γ levels in culture supernatants were measured by ELISA. Data shown are representative of two independent experiments.

At the protein level, BMDC stimulated with GLA or MPL showed similar dose-dependent increases in IL-12p40, TNF, and IL-6 pro-inflammatory cytokines (Fig. 3A). LPS appeared 10-100-fold more potent than GLA and MPL, while no background responses were observed with the buffer control (AF). In addition to the production of cytokines and chemokines, a hallmark of DC activation is the up-regulation of co-stimulatory molecules on the cell surface and increased antigen presentation capability. Therefore, we characterized BMDC surface expression of MHC class II, CD40 and CD86 in response to GLA stimulation in two mouse strains (C57BL/6, BALB/c) by flow cytometry. GLA induced a dose-dependent increase in CD40 and CD86 on the cell surface of DC from both mouse strains, while it failed to further increase MHC class II on these cells (Fig. 3B). No major differences were observed between GLA, MPL and LPS (tested at 10 ng/mL) potency at regulating the three surface molecules tested. Finally, to address the effect of GLA on DC-mediated antigen presentation, **we used a novel recombinant subunit vaccine antigen called ID83 which combines three antigens linked in tandem belonging to families of** ***Mycobacterium tuberculosis*** **proteins associated with virulence (Rv2608, Rv3620) or latency (Rv1813), and which when combined with various TLR agonists have individually and fused together elicited protection against aerosolized** ***M. tuberculosis*** [**22]**, [29****] **.** BMDC were pulsed with or without the **model antigen** ID83 in the absence or presence of 1–20 ng/mL of TLR4 agonist, and were then cultured for 48 h with CD4+ T cells purified from the splenocytes of ID83 immunized mice. DC pulsed with 50 ng/mL of ID83 in the presence of 1 ng/ml GLA induced significantly (*P*<0.05) higher IFN-γ secretion by CD4 T cells, while the addition of similar concentrations of MPL or LPS had no effect (Fig. 3C). No effect of GLA was observed at higher antigen concentrations (500–5000 ng/mL) (data not shown). This finding further supports the role of GLA in enhancing antigen presentation by DC, especially at low antigen concentration.

### Rapid Activation of Dendritic Cells to Synthetic GLA

**Figure 4:**
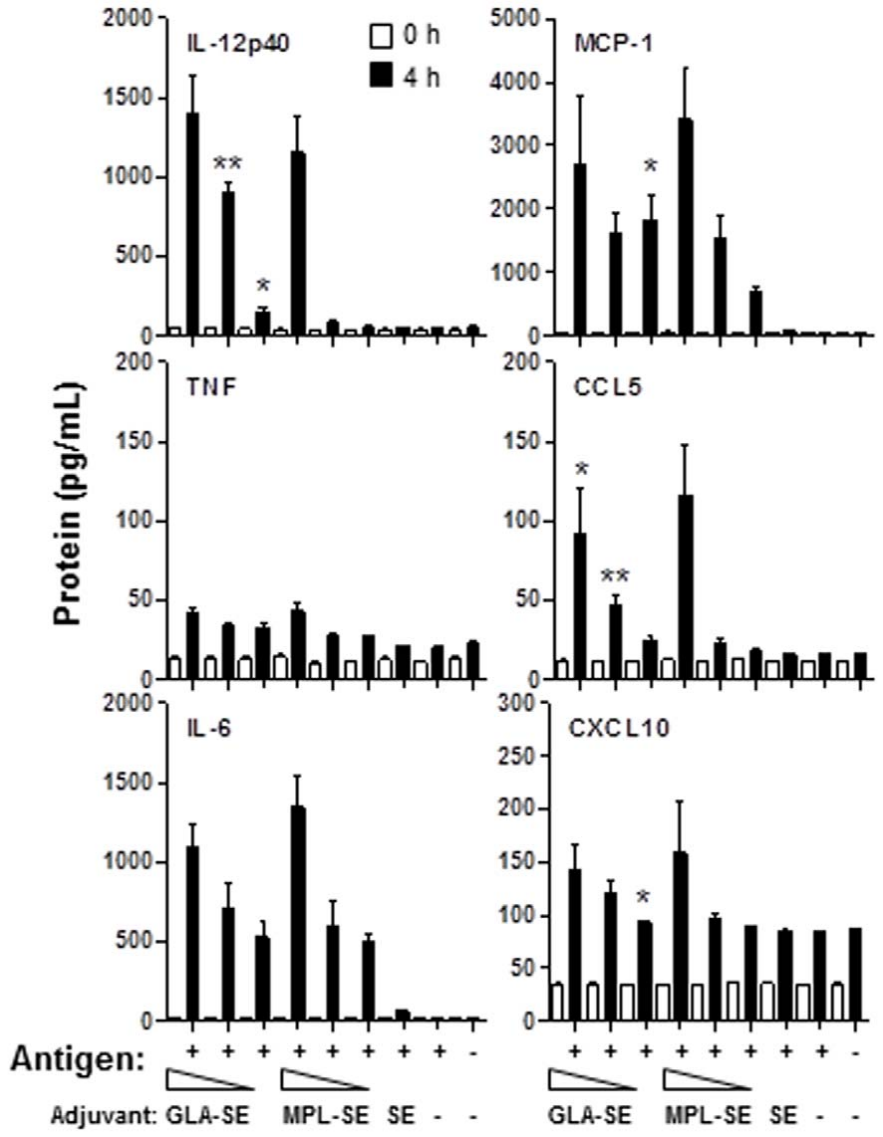
Rapid activation of dendritic cells in response to GLA. C57BL/6 mice (n = 6) were immunized 3x, 2 wks apart with ID83 antigen co-administered with 1, 5 or 20 µg of GLA-SE or MPL-SE. Control groups included saline, ID83, and ID83+SE. (A) Mice were bled before (time 0) and 4 h after the first injection. Innate cytokine levels of IL-12p40, TNF, IL-6, MCP-1, CCL5 and CXCL10 were determined by Luminex.

We next characterized the *in vivo* local and systemic effects of the GLA adjuvant in a squalene-based oil-in-water emulsion (GLA-SE), when co-administered with ID83 [22]. C57BL/6 mice were injected with ID83 i.m. three times two weeks apart with adjuvant doses of 1, 5 and 20 µg of GLA-SE or MPL-SE along with relevant controls, including antigen with SE alone. Because stimulation of cytokine and chemokines secretion plays a vital role in recruitment and activation of innate and effector immune cells, such as monocytes, macrophages, DC and T cells, we determined serum innate cytokine and chemokine responses 4 h following the first injection. Both GLA-SE and MPL-SE induced high cytokine (TNFα, IL-12p40, IL-6) and chemokine (MCP-1, CCL5, CXCL10) responses compared to saline, ID83 and ID83+SE controls (Fig. 4). These responses were dose-dependent and showed the highest responses at 20 µg TLR4 agonist immunization. There were no statistical differences between GLA-SE and MPL-SE induced TNF or IL-6 responses, while higher levels of IL-12p40 were measured in response to 5 µg and 1 µg of GLA-SE compared to equivalent amounts of MPL-SE (*P*<0.01 and *P*<0.05, respectively). Similarly, an increase in levels of MCP-1, CCL5 and CXCL10 were observed with 5 µg and/or 1 µg of GLA-SE compared to MPL-SE. Levels of IL-10, a regulatory cytokine, was below the limit of detection for all groups tested (data not shown).

### GLA Promotes a T_H_1-type Response

**Figure 5:**
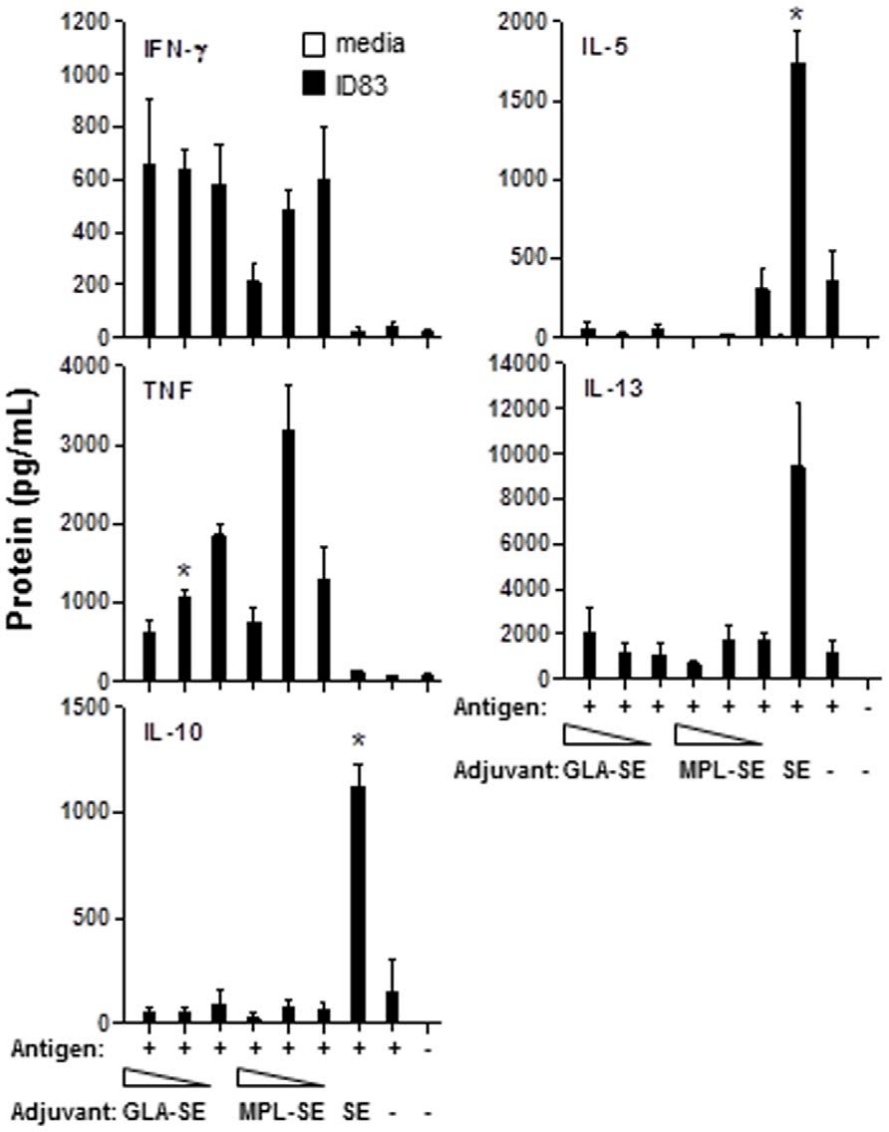
GLA promotes a T_H_1-type response. C57BL/6 mice (n = 6) were immunized 3x, 2 wks apart with ID83 antigen co-administered with 1, 5 or 20 µg of GLA-SE or MPL-SE. Control groups included saline, ID83, and ID83+SE. Splenocytes were harvested 10 days following the third immunization and stimulated in vitro with ID83 for 72 h. Levels of T_H_1 (IFN-γ, TNF), T_H_2 (IL-5, IL-13) and regulatory (IL-10) cytokines in culture supernatants were determined by multiplex ELISA (Luminex). Data shown are mean ± SD of triplicate wells, and are representative of two independent experiments. * *P*<0.05, ** *P*<0.01.

To determine the effect of GLA on adaptive immune T cell responses, **mice were injected with ID83 i.m. three times two weeks apart with adjuvant doses of 1, 5 and 20** µ**g of GLA-SE or MPL-SE along with relevant controls**, and splenocytes were collected from mice ten days after the third immunization and incubated with or without ID83 *in vitro* for 72 h. The levels of T_H_1/T_H_2 and regulatory cytokines (IFN-γ, TNF/IL-5, IL-13 and IL-10, respectively) in culture supernatants were determined by using a Luminex assay. As previously observed with a different vaccine [22], injection of the antigen in SE resulted in strong T_H_2-biased immune responses characterized by high levels of IL-5 and IL-13 (*P*<0.05 compared to ID83 alone) induction and the absence of IFN-γ and TNF (Fig. **5**). In contrast, GLA-SE and MPL-SE had measurable adjuvant effects on the priming of ID83-specific T cells, with a strong skewing towards a T_H_1-type immune response characterized by elevated levels of IFN-γ and TNF and absence of the T_H_2 cytokines IL-5 and IL-13, as compared with SE alone (Fig. **5**). There was significantly less TNF observed with GLA at 1 µg compared to (*P*<0.05). Furthermore, the regulatory cytokine IL-10 was detected only in mice that were immunized with ID83 formulated with SE, but not GLA-SE or MPL-SE (Fig. **5**)

### Enhanced Potency of GLA on Human Innate Immune Responses

**Figure 6:**
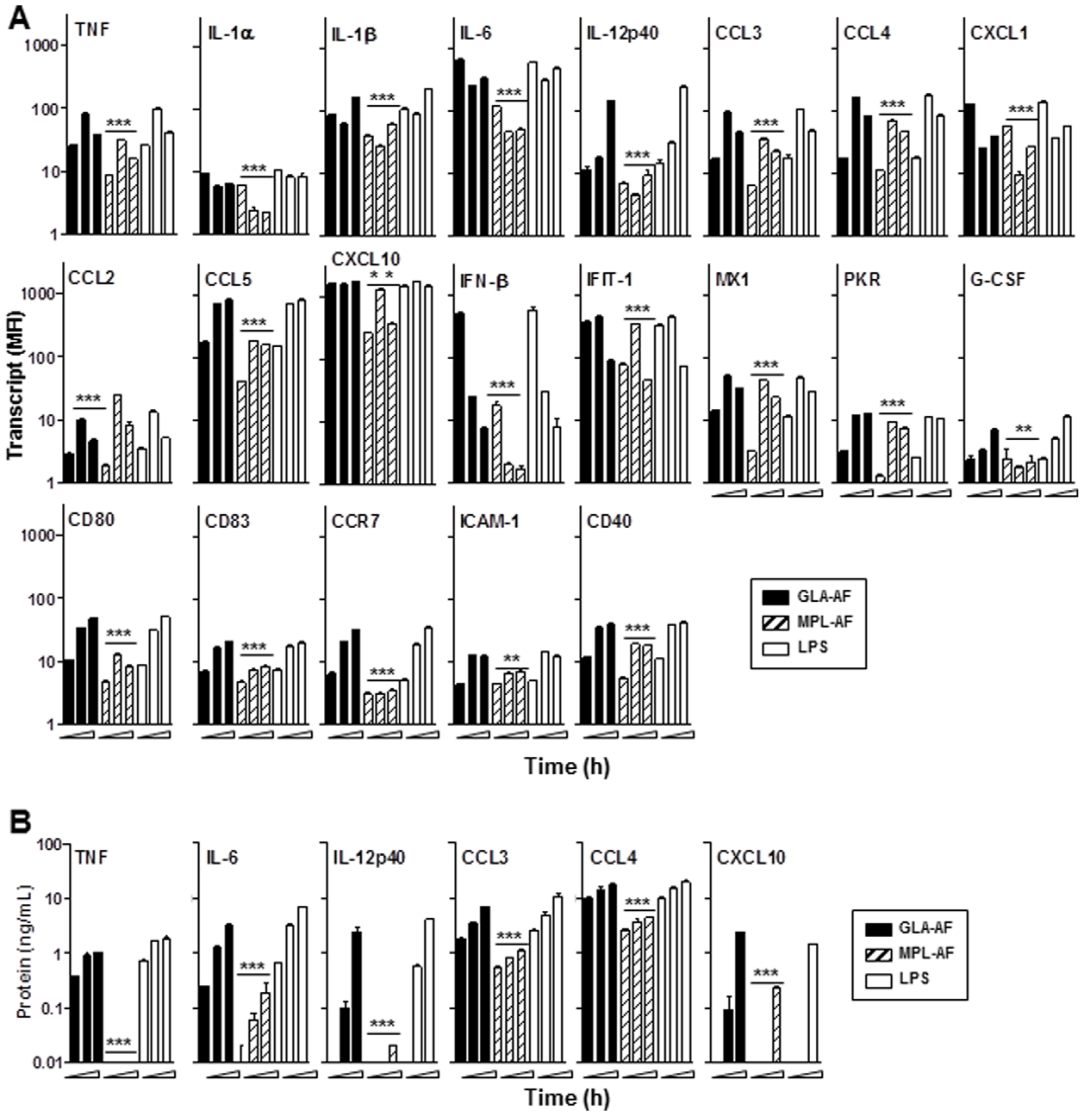
Kinetic of human DC gene expression in response to TLR4 agonist stimulation. DC were stimulated with 1 µg/mL of GLA-AF, MPL-AF, or LPS. (A) RNA transcripts for a panel of MyD88- and/or TRIF-dependent genes (upper panel), TRIF-dependent only genes (middle panel), or genes associated with DC maturation (lower panel) were captured after 2, 4, and 8 h of stimulation using the QuantiGene multiplex assay. (B) Protein levels were determined after 4, 8, and 24 h stimulation for a subset of molecules in culture supernatants by ELISA. Data shown are the mean ± SD of duplicate wells, and are representative of DC from two blood donors. Difference significance between GLA-AF and MPL-AF groups at a given time point were determined by unpaired Student's *t*-Test, *P*<0.05 (*).

Structure-function analysis of synthetic lipid A derivatives indicates that the length and number of acylated chains are critical for signaling through TLR4 and activating human, but not mouse, innate immune responses [30], [31], [32]. Therefore, we characterized the activity of hexaacylated lipid A GLA on human monocyte-derived DC at the RNA and protein levels, and compared it to MPL, a mixture of penta, hexa and hepta-acyl chains. Monocyte-derived DC from healthy human donors were stimulated with 1 µg/mL of GLA, MPL and LPS, and RNA transcript levels of MyD88- and/or TRIF- dependent or TRIF-dependent only genes were quantified using the QuantiGene assay. Lower levels of RNA transcripts were observed in human DC stimulated with MPL when compared to GLA or LPS, for genes of the MyD88- and/or TRIF-dependent pathway (Fig. **6**A top panel), while no differences in transcript levels were seen in mouse BMDC (Table 1). Compared to BMDC, GLA failed to induce IL-1α, and up-regulated IL-1β, IL-12p40, TNF, CCL3, and CCL4 in human DC. Genes activated through TRIF only showed RNA transcripts levels mostly similar between GLA and MPL, with the exception of lower levels of CCL5 in response to MPL activation in human DC (Fig. **6**A middle panel). TLR4 agonists induced higher levels of CCL5, CXCL10, IFIT-1, and MX1 transcripts in human compared to mouse DC (Table 1). To ensure that differences in transcript levels observed in response to GLA and MPL activation were not due to differences in kinetics, human DC were incubated with the TLR4 agonists for 2–8 h. Transcript levels of genes from the MyD88- and/or TRIF-dependent axis at all three time points were higher in response to GLA activation when compared to the corresponding time points in response to MPL (Fig. **6**A). Transcripts levels of TRIF-dependent only CCL5 and IFN-β were higher in the GLA groups at all time points, however the highest IFN-β levels were observed early after stimulation (2 h) and declined at 4 and 8 h. Increased levels of CXCL10, IFIT-1, MX1, and PKR in response to GLA were observed only at 2 h when compared to MPL. At the protein level, increases in molecule concentration were time-dependent (4 h<8 h<24 h), and GLA versus MPL profiles correlated with the RNA results (Fig. **6**B).

**Table 1:** DC gene expression in response to TLR4 stimulation.

	Mouse			Human		
*MyD88- and/or TRIF-dependent*	GLA^a^	MPL	LPS	GLA	MPL	LPS
IL-1α	59±8^b^	59±3	118±8	6±0	3±0	8±0
IL-1β	15±1	16±1	24±2	62±4	28±0	91±2
IL-6	209±4	221±10	298±5	246±4	47±0	313±7
IL-12p40	9±0	7±0	13±0	18±1	5±0	31±2
TNF	13±1	14±1	25±0	81±3	31±1	98±3
CCL3 (MIP-1α)	8±1	9±1	7±1	96±2	35±2	105±5
CCL4 (MIP-1β)	38±3	42±5	39±3	166±3	71±1	179±5
CXCL1 (KC)	23±1	21±0	84±2	25±1	10±1	37±1
***TRIF-dependent only***						
CCL2 (MCP-1)	8±0	8±0	7±1	10±1	25±1	23±1
CCL5 (RANTES)	ND^c^	ND	ND	719±9	185±5	748±1
CXCL10 (IP-10)	57±4	66±10	56±5	1509±52	1242±74	1658±94
IFNβ	55±5	47±4	48±0	24±1	2±0	28±1
IFIT-1	77±9	85±2	70±0	449±28	353±2	436±23
MX1	ND	ND	ND	52±2	44±0	49±1
PKR	13±1	13±0	11±0	12±0	10±0	12±0
G-CSF	76±7	80±9	201±5	3±0	2±0	5±1

a Mouse bone-marrow derived and human monocyte-derived DC (5×10^4^) were incubated with 1 µg/mL of MPL, GLA or LPS for 4 h in 96-well round bottom plates. Transcript expression was determined using the QuantiGene multiplex assay. Data shown is representative of two independent experiments.

b mRNA fold increase upon TLR-4 stimulation compared to buffer control (and normalized to GAPDH). Mean ± SD are shown.

c ND, not done.

**Figure 7:**
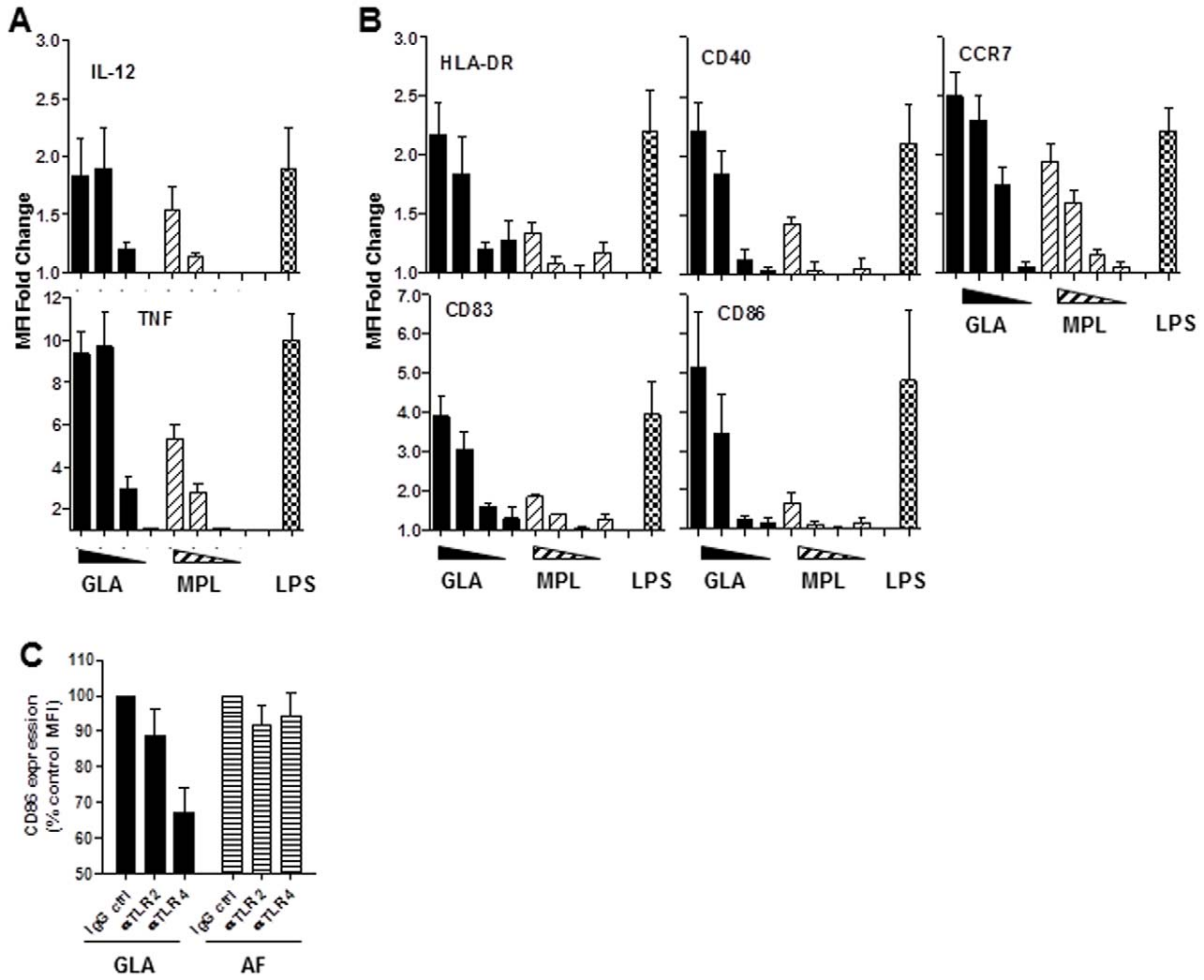
Dose-dependent activation and maturation of human DC in response to GLA stimulation. Human monocyte-derived DC were incubated with 1, 10, 100, and 1000 ng/mL of GLA-AF or MPL-AF, or 1000 ng/mL of LPS. Levels of cell surface co-stimulatory molecules and intracellular cytokines were determined by flow cytometry. (A) IL-12p70 and TNF mean fluorescence fold (MFI) change over AF control at 12 h post-stimulation. (B) HLA-DR, CD40, CCR7, CD83, and CD86 co-stimulatory molecules MFI change over AF control at 48 h post-stimulation. (C) CD86 expression on DC stimulated with 100 ng/mL GLA-AF or -AF for 48 h in the presence of 1 µg/mL of anti-TLR-2, anti-TLR4, or IgG2a isotype control antibody. Data shown are the mean ± SD (n = 3, DC cultures from different blood donors), and representative of two independent experiments.

Transcript levels of markers associated with DC maturation, such as CD40, CD80, CD83, CCR7, and ICAM-1, were also higher in response to GLA compared to MPL (Fig. **6**A lower panel). TLR4 agonist dose-dependency was further addressed by characterizing human DC activation (IL-12p40, TNF) and maturation (HLA-DR, CD40, CD83, CD86, CCR7) markers by a range of doses (1 to 1000 ng/mL) using flow cytometry. Increased mean fluorescence intensity (MFI) expression of all the molecules tested in response to GLA stimulation was dose-dependent, and at least 10-fold more MPL was needed to achieve the same level of protein up-regulation (Fig. **7**A–B). TLR-blocking experiments confirmed that GLA signaled through TLR4, but not TLR2 in human DC (Fig. **7**C).

Finally, we demonstrated that GLA activated human PBMC in a dose-dependent manner for the secretion of cytokines such as IL-12p40, TNF, IL-6, IL-1β, IL-10, IL-17 and IFN-γ (Fig. S2A) and chemokines such as CCL4, CCL5, CCL7, and CXCL10 (Fig. S2B). As with human DC, PBMC showed higher responses to GLA than MPL. Both induced lower cytokine/chemokine levels than LPS at comparable agonist concentration, with the exception of CXCL10.

## Discussion

The capacity of host DC to mount an effective early innate immune response to adjuvants may significantly impact the overall immunogenicity and efficacy of vaccines. Derivatives of LPS, a TLR4 agonist, such as purified detoxified monophosphoryl lipid A (MPL®) and synthetic GLA are either approved or being tested in clinical trials as novel vaccine adjuvants for use in humans [10], [11], [33] (Tom Dubensky, personal communication). **Numerous prior studies have corroborated our results and reported** that structural differences such as the degree of phosphorylation, and number, position and length of acyl chains can dramatically affect the signaling outcome [30], [32], [34], [35], [36], [37]. The studies presented here provide insight into mouse and human DC innate immune response profiles to the synthetic hexaacylated lipid A GLA, at the mRNA and protein levels. We demonstrate that, while GLA and a MPL purified from *S. minnesota* R595 induced similar dose- and time-dependent responses in mouse BMDC, GLA was 10–100-fold more active than MPL, a predominantly pentaacylated lipid A, on human monocyte-derived DC. Finally, we show that when administered to mice in a stable oil-in-water emulsion (SE), GLA-SE induced strong systemic innate responses and priming of antigen-specific T_H_1 cells.

LPS molecules are complex glycolipids present in the outer membrane of Gram-negative bacteria. The lipid A domain of LPS consists of a disaccharide with acyl chains of variable length and number and is mostly responsible for its endotoxic activity by inducing pro-inflammatory cytokines and type I IFN through the TLR4/MD-2/MyD88 and TLR4/MD-2/TRIF pathways, respectively[13], [31], [38]. Detoxification of LPS from *S. minnesota* R595, for example, was achieved by the removal of the core carbohydrate group, the phosphate from the reducing-end glucosamine, and the acyl chain from the 3′-position of the disaccharide backbone [5]. The resulting molecule MPL® retained only ∼0.1% of the inflammatory toxicity of its parent LPS molecule [7], [39], and was initially shown to preferentially signal through the TRIF rather than the MyD88 pathway in mouse macrophages [35], though subsequent studies demonstrated that MPL signals through both MyD88 and TRIF[40]. GLA, which also lacks the second phosphate group, showed reduced endotoxicity compared to LPS, and induced levels of BMDC innate immune responses comparable to MPL, respectively (Fig. 2–3, S2, Table 1). Similarly to MPL [35], GLA signaled less efficiently than LPS through the MyD88/MAL-dependent pathway, but fully retained the ability to signal through TRIF/TRAM.

Depending on the mammalian species, the activities of different types of lipid A can differ markedly at the TLR4/MD-2 complex. While LPS and hexaacylated lipid A function as agonists in most species, tetraacylated lipid IVa and Eritoran are agonists in mice, and antagonists in humans and non-human primates [30], [31]. Furthermore, a synthetic pentaacylated lipid A has also been shown to lack bioactivity on HEK293T cells transfected with human TLR4/MD-2 and on human PBMC [32]. Differences in crystal structure of human TLR4/MD-2/LPS [36] and TLR4/MD-2/lipid IVa [41] or TLR4/MD-2/Eritoran [42] complexes recently shed light on the molecular and spatial interactions between TLR4/MD-2 with agonist, and antagonists, respectively. These studies showed that four acyl chains (Eritoran, lipid IVa) completely filled the MD-2 pocket, but failed to induce receptor dimerization and lacked agonistic activity [34], [41]. In contrast, in the case of the hexaacylated LPS from *E. coli*, accommodating a fifth acyl chain in the MD-2 pocket resulted in displacement of the glucosamine backbone and reposition of the phosphate groups which interact with positively charged residues on the TLR4/TLR4* dimers. The sixth acyl chain also interacts with the TLR4/TLR4* dimers and further contribute to the stabilization and agonistic activity of the complex [36]. Consistent with these previous observations, we show here that the pure hexaacylated lipid A GLA was bioactive on human DC and PBMC, resulting in DC innate immune responses, maturation and enhanced antigen-presentation, while the predominantly pentaacylated MPL was 10 – 100-fold less effective (Fig. **6** **,** **7**, and S2). Unlike a synthetic pentaacylated molecule which was completely inactive [32], MPL retained some agonistic activity likely due to the presence of some hexaacylated molecules as shown by HPLC and mass spectrometry (Fig. 1A–B). Rallabhandi *et al*. reported a 20 – 90% decreased cytokine level by adherent PBMC in response to *Shigella flexneri* 2a (a mixture of hexa, penta, and predominantly tetraacylated lipid A) than to *E. coli* (predominantly hexaacylated lipid A) LPS [32] further illustrating the importance of using hexaacylated lipid A derivatives when it comes to humans. Because GLA lacks one phosphate group, it still remains several orders of magnitude less endotoxic than its parent molecule LPS (Fig. S2), and is safer to use in humans. TLR4 polymorphisms resulting in decreased cytokine production in response to LPS [32] have been reported to affect 6–10% of the population. However, it remains to be shown if responses to GLA would also be decreased in these individuals.

Protective immunity to tuberculosis (TB) is conferred by IFN-γ producing T_H_1 CD4 and effector CD8 T cells [43]. Recently, TLR4 agonists, including GLA, have been shown to induce potent T_H_1-biased immune responses [20], [22], [23], [24]. In the current study, we observed a correlation between systemic innate responses, enhanced antigen-presentation and T_H_1 priming. Particular, innate pro-inflammatory cytokines and chemokines that are involved in recruitment of antigen presenting cells and effector T cells are enhanced following immunization with ID83 (TB fusion vaccine antigen) formulated with GLA-SE. For example, IL-12p40 is a chemoattractant for recruiting macrophages to the site of infection, but also promotes migration of activated DC [44], supporting our findings that GLA enhances T_H_1-biased immune responses.

Altogether, the data presented here support the adjuvant activity of GLA both *in vitro* and *in vivo*, and provide mechanistic evidence for the protective immune responses elicited by GLA-containing vaccines in animal models of tuberculosis and leishmaniasis [22], [24]. Furthermore, GLA presents the advantages of a pure synthetic component, stable in aqueous and oil-in-water emulsions, fully active on human DC and PBMC, that has already been scaled up and cGMP manufactured, and was recently tested in a Phase I clinical trial for seasonal influenza **where it was shown to be well tolerated and immunogenic** (personal communication Tom Dubensky).

## Supporting Information

Figure S1 **BMDC gene expression in response to TLR4 agonist stimulation.** BMDC were stimulated for 2, 4, and 8 h with 1 µg/mL of GLA-AF, MPL-AF, or LPS. RNA transcripts for a panel of MyD88- and/or TRIF- or TRIF-dependent only genes were captured using the QuantiGene multiplex assay. Data shown are the mean ± SD of duplicate wells, and are representative of three independent experiments.
(TIF)

Figure S2 **PBMC immune responses to TLR4 agonists.** PBMC from healthy human donors were stimulated with 0.001 to 1000 ng/mL of TLR4 agonists for 24 h. Multiplex assays were then performed on sample supernatants, focusing on cytokines and chemokines that influence T_H_ cell responses. Data shown are the mean ± SD of duplicates wells, and are representative of three independent experiments. (A) Secretion of cytokines. (B) Secretion of chemokines.
(TIF)
