# Supplementary figures and images for: Development and Characterization of Synthetic Glucopyranosyl Lipid Adjuvant System as a Vaccine Adjuvant
Source: PLoS One. 2011 Jan 26;6(1):e16333. doi: 10.1371/journal.pone.0016333 (PMC3027669; doi:10.1371/journal.pone.0016333)

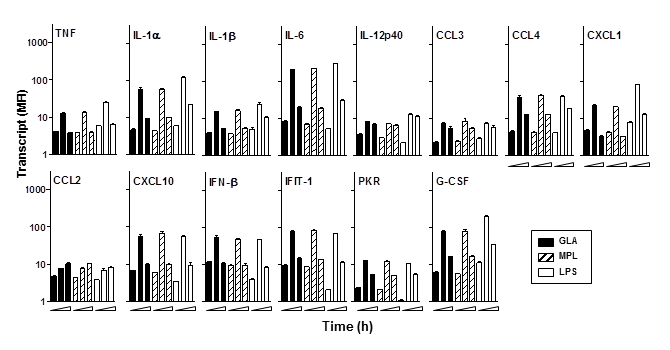

Supplement: Figure S1 — BMDC gene expression in response to TLR4 agonist stimulation. BMDC were stimulated for 2, 4, and 8 h with 1 µg/mL of GLA-AF, MPL-AF, or LPS. RNA transcripts for a panel of MyD88- and/or TRIF- or TRIF-dependent only genes were captured using the QuantiGene multiplex assay. Data shown are the mean ± SD of duplicate wells, and are representative of three independent experiments. (TIF) [file pone.0016333.s001.tif]

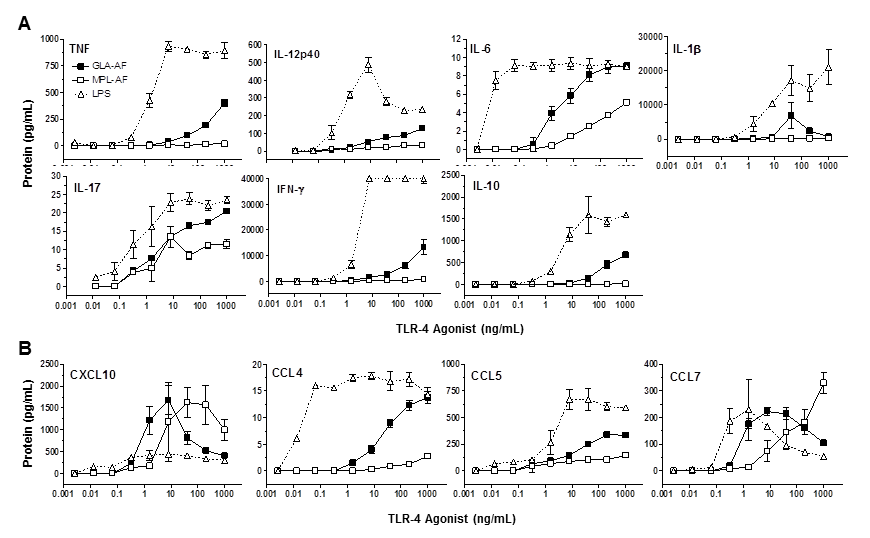

Supplement: Figure S2 — PBMC immune responses to TLR4 agonists. PBMC from healthy human donors were stimulated with 0.001 to 1000 ng/mL of TLR4 agonists for 24 h. Multiplex assays were then performed on sample supernatants, focusing on cytokines and chemokines that influence TH cell responses. Data shown are the mean ± SD of duplicates wells, and are representative of three independent experiments. (A) Secretion of cytokines. (B) Secretion of chemokines. (TIF) [file pone.0016333.s002.tif]
